# Contemporary outcomes of a DCB-based strategy with selective stent implantation for femoropopliteal artery lesions: results from the REAL-LEAD registry

**DOI:** 10.1186/s42155-026-00744-1

**Published:** 2026-07-30

**Authors:** Takafumi Fujita, Makoto Sugihara, Kaori Mine, Kenji Ogata, Shinya Kobata, Shotaro Furukawa, Takumi Yoshiga, Yuta Ishizaki, Kensuke Oe, Atsushi Kakazu, Ryosuke Yanari, Ryoichi Kyuragi, Shoichiro Furukawa, Saburo Kusumoto, Ryohei Akashi, Eiji Karashima, Hideki Doi, Shin-ichiro Miura

**Affiliations:** 1 Department of Cardiology, Fukuoka University School of Medicine, 7-54-1 Jonan, Fukuoka, 814-0180 Japan; 2 Department of Cardiology, Miyazaki Medical Association Hospital, 1173 Arita, Miyazaki, 880-2102 Japan; 3 Present address: Department of Cardiology, Okinawa Chubu Tokusyukai Hospital, 801 Chuto, Okinawa, 901-2393 Japan; 4 Department of Cardiology, Kumamoto Rosai Hospital, 1670 Takehara-machi Yatushiro, Kumamoto, 866-0826 Japan; 5 Department of Internal Medicine, Division of Cardiovascular Medicine, Kurume University School of Medicine, 67 Asahi-Machi, Kurume, 830-0011 Japan; 6 Present address: Department of Cardiology, Ishizaki Heart Clinic, 245 Omuta, Fukuoka, 837-0901 Japan; 7 Department of Cardiology, St. Mary’s Hospital, 422 Kurume, Fukuoka, 830-8543 Japan; 8 Department of Cardiology, Yuuai Medical Center, 50-5 Yone, Tomigusuku-City, , Okinawa 901-0224 Japan; 9 Department of Cardiology, Fukuoka Seisyukai Hospital, 4-11-8 Kasuya, Fukuoka, 811-2316 Japan; 10 Department of Surgery, Saiseikai Karatsu Hospital, 817 Motohata, Karatsu, Saga 847-0852 Japan; 11 Department of Cardiology, Aso Iizuka Hospital, 3-83 Iizuka, Fukuoka, 820-0018 Japan; 12 Department of Cardiology, Saiseikai Fukuoka General Hospital, 1-3-46 Tenjin, Chuo-ku, Fukuoka, 810-0001 Japan; 13 Department of Cardiology, Nagasaki Harbor Medical Center, 6-39 Shinchi-Machi, Nagasaki, 850-8555 Japan; 14 Department of Cardiovascular Medicine, Nagasaki University Graduate School of Biomedical Sciences, 1-7-1 Sakamoto, Nagasaki, 852-8501 Japan; 15 Department of Cardiology, Shimonoseki City Hospital, 1-13-1 Shimonoseki, Yamaguchi, 750-8520 Japan

**Keywords:** Lower extremity arterial disease (LEAD), Endovascular treatment (EVT), Femoropopliteal artery lesions (FPA lesions), Drug-coated balloon (DCB)

## Abstract

**Background:**

A drug-coated balloon (DCB)-based “leave-nothing-behind” strategy has become a predominant approach for femoropopliteal artery (FPA) lesions in contemporary clinical practice. However, despite the intention to complete procedures with DCB alone, stent implantation is frequently required because of severe dissection, elastic recoil, or suboptimal lesion preparation. Whether DCB angioplasty combined with selective stent implantation compromises 1-year primary patency compared with DCB monotherapy remains uncertain.

**Methods:**

In this prospective multicenter registry, 310 limbs in 277 patients undergoing endovascular therapy for FPA lesions between September 2021 and December 2022 were enrolled. Thirty-seven limbs were excluded if they had isolated common femoral artery lesions (*n* = 8), were treated with bare nitinol stents or stent grafts (*n* = 25), or were treated with plain old balloon angioplasty alone (*n* = 4). A total of 273 limbs in 254 cases were included in the primary outcome analysis. Patients were categorized into three groups: the DCB group (balloon angioplasty with DCB alone), the drug-eluting stent (DES) group (implantation of Eluvia™ or Zilver PTX™), and the Combination group (DCB angioplasty combined with DES). The primary endpoint was 1-year primary patency. Secondary endpoints included freedom from clinically driven target lesion revascularization (CD-TLR) and clinically driven target vessel revascularization (CD-TVR), which were assessed using Kaplan–Meier analysis. Factors associated with loss of primary patency were evaluated using Cox regression analysis.

**Results:**

DCB monotherapy was performed in 78.3% of cases, DESs in 10.9%, and combination therapy in 10.6%. The overall 1-year primary patency rate was 84.0%, with no significant differences among strategies (DCB: 84.1%, DES: 84.6%, Combination: 82.8%; log-rank *p* value = 0.91). Freedom from CD-TLR and CD-TVR at 1 year was 88.7% and 93.8%, respectively. Chronic limb-threatening ischemia and history of revascularization were independently associated with loss of primary patency. Combination therapy was not associated with an increased risk of loss of primary patency.

**Conclusion:**

A DCB-based strategy with selective scaffolding may achieve acceptable patency outcomes.

**Graphical Abstract:**

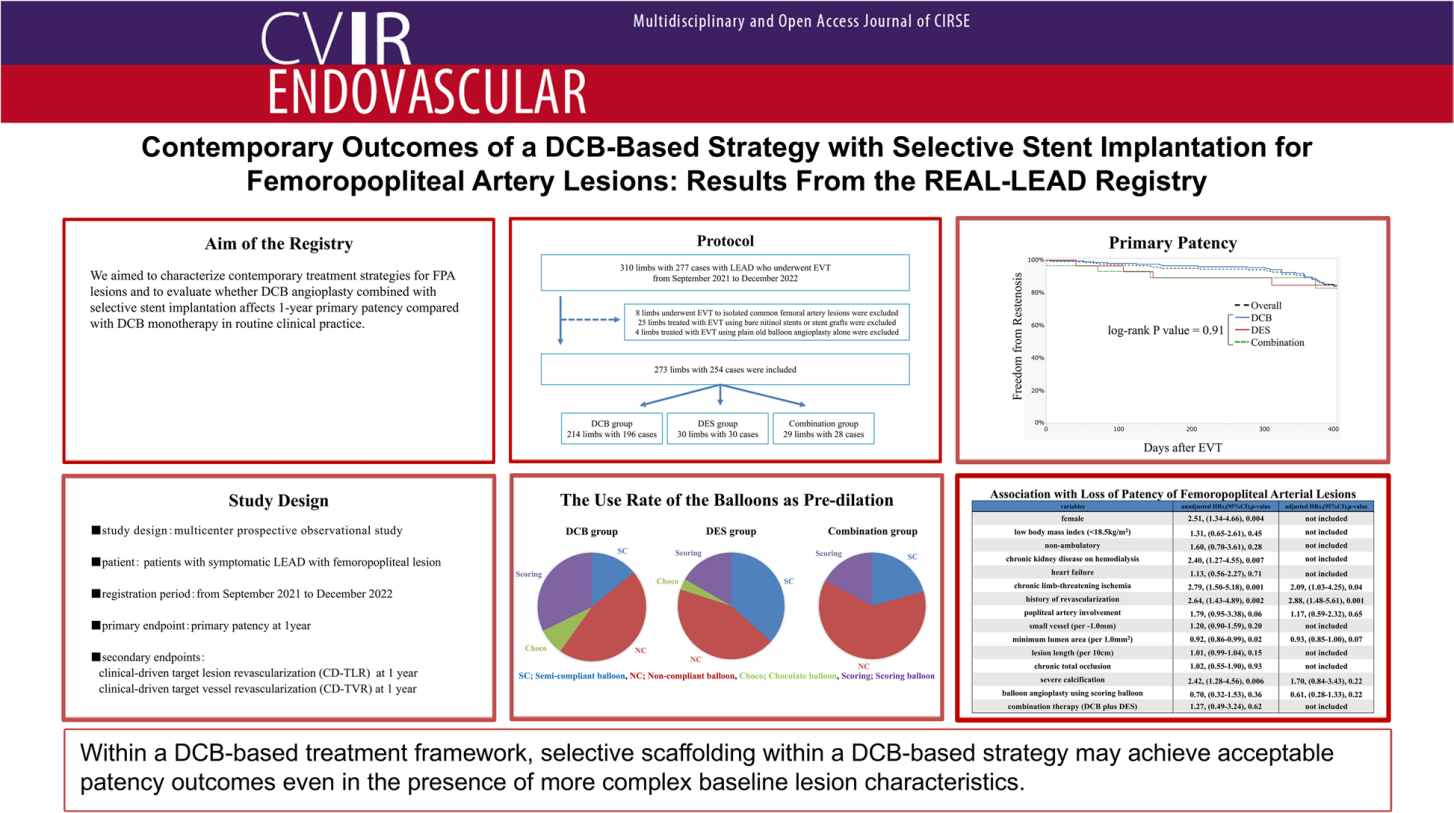

**Supplementary Information:**

The online version contains supplementary material available at 10.1186/s42155-026-00744-1.

## Introduction

Endovasculartherapy (EVT) is strongly recommended for femoropopliteal artery (FPA) lesions in patients with lower extremity arterial disease (LEAD), particularly for lesions shorter than 25 cm, according to the current guidelines [1–3]. Drug-coated balloons (DCBs) using paclitaxel have demonstrated superiority over conventional balloon angioplasty in reducing restenosis and the need for repeat revascularization [4, 5]. Furthermore, previous studies have reported acceptable outcomes of DCB even in complex lesions, including chronic total occlusion (CTO), long lesions, and in-stent restenosis [6–8].

Based on these findings, a DCB-based “leave-nothing-behind” strategy has become widely adopted in Japan. However, in real-world practice, achieving a DCB-only strategy is not always feasible. Severe calcification, elastic recoil, and flow-limiting dissection may compromise acute results despite adequate lesion preparation [8–11]. In such cases, adjunctive stent implantation is often required to secure sufficient luminal gain and procedural success. Paclitaxel-eluting stents as well as non–drug-eluting scaffolding devices, including stent grafts and bare nitinol stents, are therefore selected according to lesion characteristics.

Although prior studies [4, 5, 12–15] have reported favorable outcomes with individual devices, a previous report has suggested that combined DCB angioplasty and stenting may be associated with inferior patency [16]. Nevertheless, in routine practice, DCB angioplasty combined with selective stent implantation is frequently performed in anatomically complex lesions. Whether such a strategy compromises mid-term patency compared with DCB monotherapy remains uncertain. Therefore, using data from the REAL-LEAD registry, we aimed to characterize contemporary treatment strategies for FPA lesions and to evaluate whether DCB angioplasty combined with selective stent implantation affects 1-year primary patency compared with DCB monotherapy in routine clinical practice.

## Method

### Study design and patient population

The REAL-LEAD registry is a multicenter, prospective, observational study conducted at 16 institutions in Japan. Eligible patients were ≥ 20 years of age and had symptomatic lower extremity arterial disease corresponding to Rutherford classification 2–5. Lesions located in the superficial femoral artery (SFA) and/or popliteal artery segments (P1–P3) were included. Patients with an estimated life expectancy of less than 1 year were excluded. In addition, patients scheduled for surgical bypass or major amputation (amputation proximal to the ankle joint) within 30 days after EVT were excluded. Ischemia was defined as an ankle–brachial index (ABI) ≤ 0.90 at rest. In patients with ABI > 0.90, ischemia was confirmed by additional objective testing, including a decrease of ≥ 20 mmHg in ankle systolic pressure or ≥ 20% reduction in ABI during exercise testing, a toe–brachial index < 0.70, transcutaneous oxygen pressure < 60 mmHg, or skin perfusion pressure < 60 mmHg. Target lesions were defined as de novo or restenotic FPA lesions with ≥ 50% diameter stenosis assessed by angiography, contrast-enhanced computed tomography, or magnetic resonance imaging. Hemodynamic stenosis was defined as a peak systolic velocity ratio (PSVR) > 2.4 on duplex ultrasound.

**Fig. 1:**
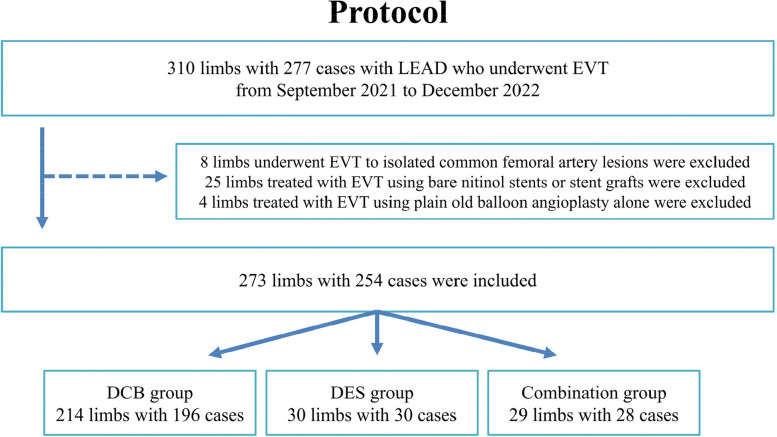
Flowchart of the patient enrollment process. Abbreviations: LEAD, lower extremity artery disease; EVT, endovascular therapy; DCB, drug-coated balloon; DES, drug-eluting stent

Between September 2021 and December 2022, 310 limbs from 277 consecutive patients undergoing EVT for FPA lesions were prospectively enrolled. A total of 273 limbs were included in the primary outcome analysis. 37 limbs were excluded if they had isolated common femoral artery lesions (*n* = 8), were treated with bare nitinol stents or stent grafts (*n* = 25) or underwent plain old balloon angioplasty alone (*n* = 4) (Fig. 1). Moreover, patients were categorized according to the final device strategy into three groups: the DCB group (balloon angioplasty with DCB alone), the DES group (drug-eluting stent (DES) implantation [Eluvia™ or Zilver PTX™]), and the Combination group (DCB angioplasty combined with DES).

The study protocol was approved by the local ethics committee at each participating institution. Written informed consent was obtained from all patients prior to EVT in accordance with institutional and national regulations.

### Revascularization procedure

Procedural strategies were left to the discretion of the treating operators and reflected routine clinical practice. The access site, lesion preparation techniques, and final device selection were determined by each operator. Dual antiplatelet therapy was recommended for at least 1 month after EVT according to institutional practice.

Available DCBs included IN.PACT™ Admiral™ (Medtronic, Dublin, Ireland), Lutonix™ (BD, Franklin Lakes, NJ, USA), and Ranger™ (Boston Scientific, Marlborough, MA, USA). Available drug-eluting stents included Eluvia™ (Boston Scientific) and Zilver PTX™ (Cook Medical, Bloomington, IN, USA).

Debulking devices were not reimbursed during the study period and were therefore not included in this registry.

Severity of vessel calcification was graded using the Peripheral Artery Calcium Scoring System (PACSS) [17]. The severity of dissection was assessed after pre-balloon angioplasty because, while the decision to implant a stent was made at the operator’s discretion, the extent of dissection substantially influenced that decision. The severity of dissection was classified according to the National Heart, Lung and Blood Institute (NHLBI) classification system [18].

### Outcome measurement

The primary endpoint was 1-year primary patency of the target FPA lesion, defined as freedom from restenosis. Restenosis was determined by a PSVR > 2.4 on duplex ultrasound and/or ≥ 50% diameter stenosis on contrast-enhanced computed tomography, magnetic resonance imaging, or angiography. The primary endpoint was evaluated at 1 year after EVT. Follow-up assessments were allowed within a predefined window of ± 1 months around the 1-year time point.

Secondary endpoints included procedural success, defined as the absence of major procedural complications and < 50% residual stenosis on final angiography; clinically driven target lesion revascularization (CD-TLR); and clinically driven target vessel revascularization (CD-TVR).

### Statistical analysis

Baseline characteristics were analyzed on a per-limb basis. Continuous variables are presented as mean ± standard deviation, and categorical variables as counts and percentages. Baseline characteristics and primary patency rates were compared among the three treatment groups using the Kruskal–Wallis test for continuous variables and Fisher’s exact test for categorical variables. Time-to-event outcomes, including primary patency, freedom from CD-TLR, and CD-TVR, were analyzed using the Kaplan–Meier method and compared using the log-rank test. A multivariable Cox regression model was performed to identify factors associated with loss of primary patency. Hazard ratios (HRs) with 95% confidence intervals (CIs) were calculated. Variables included in the multivariable model were selected based on clinical relevance and prior literature, including chronic limb-threatening ischemia (CLTI), history of revascularization, severe calcification (PACSS grade 4), popliteal artery involvement, minimum lumen area (MLA) and scoring balloon use. A two-sided *P* value < 0.05 was considered statistically significant.

As a supplemental analysis, a comparison between the DCB group and the Combination group was performed. Propensity score matching was conducted using the following covariates: lesion length, age, sex, CLTI, chronic kidney disease (CKD) on hemodialysis, history of revascularization, PACSS grade, ambulatory status, reference vessel diameter, and popliteal artery involvement. Propensity score matching was performed using a nearest-neighbor algorithm without replacement, with a caliper width of 0.1 standard deviations of the logit of the propensity score. Covariate balance after matching was assessed using standardized mean differences (SMDs), with an SMD < 0.1 considered indicative of acceptable balance.

All analyses were performed using JMP version 17.2 (SAS Institute, Cary, NC, USA).

## Results

The baseline patient and lesion characteristics are summarized in Tables 1 and 2. The mean age of the study population was 75.4 ± 8.9 years, and 33.3% were female. Most patients (86.0%) were ambulatory. The prevalence of type 2 diabetes mellitus and CKD requiring hemodialysis was 67.0% and 19.0%, respectively. According to the final device strategy, 214 limbs were treated with DCB alone, 30 with DES, and 29 with DCB angioplasty combined with DES. The proportions of female patients and those undergoing hemodialysis were similar among the three groups.

**Table 1:** The baseline of patient characteristics

Variables	Overall (*n* = 273)	DCB group (*n* = 214)	DES group (*n* = 30)	Combination group (*n* = 29)	*P* value
Age, years	75.4 ± 8.9	75.2 ± 8.5	77.9 ± 7.7	73.2 ± 12.4	0.25
Female, %	91 (33.3)	71 (33.1)	10 (33.3)	10 (34.4)	0.99
BMI, kg/m^2^	22.4 ± 4.4	22.5 ± 4.6	22.8 ± 3.5	21.6 ± 3.1	0.39
Current smoker, %	64 (23.4)	53 (24.7)	6 (20.0)	5 (17.2)	0.59
Fully ambulant, %	235 (86.0)	187 (87.3)	28 (93.3)	20 (68.9)	0.01
Hypertension, %	248 (90.8)	197 (92.0)	27 (90.0)	24 (82.7)	0.26
Dyslipidemia, %	198 (72.5)	161 (75.2)	19 (63.3)	18 (62.0)	0.16
Diabetes mellitus, %	183 (67.0)	143 (66.8)	21 (70.0)	19 (65.5)	0.92
Coronary artery disease, %	130 (47.6)	103 (48.1)	10 (33.3)	17 (58.6)	0.14
CKD on HD	52 (19.0)	42 (19.6)	2 (6.6)	8 (27.5)	0.11
Heart failure, %	66 (24.1)	44 (20.5)	10 (33.3)	12 (41.3)	0.02
Cerebral vascular disorder, %	44 (16.1)	33 (15.4)	4 (13.3)	7 (24.1)	0.44
CLTI, %	100 (36.6)	66 (30.8)	15 (50.0)	19 (65.5)	0.0004
Creatinine, mg/dL	1.0 ± 0.4	1.0 ± 0.4	0.9 ± 0.2	0.8 ± 0.3	0.01
eGFR, ml/min/1.73m^2^	56.3 ± 21.8	54.1 ± 20.3	61.8 ± 25.2	67.2 ± 25.0	0.03
Total-Cho, mg/dL	159.4 ± 37.9	157.2 ± 36.5	162.7 ± 47.6	173.5 ± 35.7	0.03
LDL-Cho, mg/dL	84.2 ± 29.6	82.4 ± 29.9	86.2 ± 31.8	95.0 ± 23.2	0.03
HbA1c, %	6.5 ± 1.0	6.5 ± 0.9	6.7 ± 1.1	6.3 ± 1.0	0.29
Aspirin, %	181 (66.3)	151 (70.5)	13 (43.3)	17 (58.6)	0.008
P2Y12-i, %	236 (86.4)	184 (85.9)	28 (93.3)	24 (82.7)	0.45
Cilostazol, %	49 (17.9)	39 (18.2)	6 (20.0)	4 (13.7)	0.80
OAC, %	45 (16.4)	28 (13.0)	9 (30.0)	8 (27.5)	0.01
Statin, %	197 (72.1)	157 (73.3)	19 (63.3)	21 (72.4)	0.51

Abbreviations: *DCB* drug-coated balloon, *DES* drug-eluting stent, *BMI* body mass index, *CKD* chronic kidney disease, *HD* hemodialysis, *CLTI* chronic limb-threatening ischemia, *eGFR* estimated glomerular filtration rate, *Total-Cho* total cholesterol, *LDL-Cho* low-density lipoprotein cholesterol, *P2Y12-i* P2Y12-inhibitor, *OAC* oral anticoagulant

**Table 2:** The Baseline of lesion characteristics

Variables	Overall (*n* = 273)	DCB group (*n* = 214)	DES group (*n* = 30)	Combination group (*n* = 29)	*P* value
De novo lesion, %	204 (74.7)	160 (74.7)	22 (73.3)	22 (75.2)	0.97
History of EVT, %	69 (25.3)	54 (25.2)	8 (26.6)	7 (24.1)	0.97
History of stent implantation, %	27 (9.8)	19 (8.8)	4 (13.3)	4 (13.7)	0.56
History of DCB, %	47 (17.2)	40 (18.6)	3 (10.0)	4 (13.7)	0.43
Isolated SFA, %	154 (56.4)	118 (55.1)	24 (80.0)	12 (41.3)	0.008
CFA involvement, %	12 (4.3)	11 (5.1)	0 (0)	1 (3.4)	0.42
POP.A involvement, %	115 (42.1)	92 (42.9)	6 (20.0)	17 (58.6)	0.009
Lesion length, mm	212.1 ± 114.3	203.4 ± 116.4	205.0 ± 97.6	283.6 ± 90.6	0.001
Proximal reference diameter, mm	5.5 ± 1.0	5.5 ± 0.9	5.7 ± 1.2	5.1 ± 1.0	0.09
Distal reference diameter, mm	5.0 ± 0.9	5.0 ± 0.9	5.4 ± 0.7	4.7 ± 1.0	0.01
BK poor run off (0 or 1), %	110 (40.2)	85 (39.7)	13 (43.3)	12 (41.3)	0.92
CTO, %	135 (49.4)	99 (46.2)	18 (60.0)	18 (62.0)	0.13
CTO length, mm	74.5 ± 100.4	65.5 ± 95.2	131.5 ± 122.8	82.8 ± 96.7	0.01
Severity of calcification (PACSS grade), %					0.46
0–2	175 (64.1)	141 (65.8)	20 (66.6)	14 (48.2)	
3	26 (9.5)	19 (8.8)	3 (10.0)	4 (13.7)	
4	72 (26.3)	54 (25.2)	7 (23.3)	11 (37.9)	
TASC Ⅱ classification (C or D), %	139 (50.9)	99 (46.2)	20 (66.6)	20 (68.9)	0.01

Abbreviations: *DCB* drug-coated balloon, *DES* drug-eluting stent, *EVT* endovascular therapy, *SFA* superficial femoral artery, *CFA* common femoral artery, *POP.A* popliteal artery, *BK* below the knee, *CTO* chronic total occlusion, *PACSS* Peripheral Artery Calcium Scoring System, *TASC* Trans-Atlantic Inter-Society Consensus

Regarding clinical presentation, 63.4% of patients had intermittent claudication, whereas 38.5% presented with rest pain, gangrene, or refractory ulcer. CLTI was more frequent in the DES and Combination groups than in the DCB group. Laboratory findings and medication profiles are detailed in Table 1. Among non-dialysis patients, the mean serum creatinine level was 1.0 ± 0.4 mg/dL, and the estimated glomerular filtration rate was 56.3 ± 21.8 mL/min/1.73 m^2^. The mean low density lipoprotein-cholesterol level was 84.2 ± 29.6 mg/dL, and the mean HbA1c was 6.5 ± 1.0%.

Most lesions (74.7%) were de novo, whereas 25.3% had a history of revascularization. The proportion of history of revascularization was comparable across groups. Lesions were located exclusively in the SFA in 56.4% of cases, while 4.3% involved the common femoral artery and 42.1% involved the popliteal artery. The mean lesion length was 212.1 ± 114.3 mm. The proximal and distal reference vessel diameters were 5.5 ± 1.0 mm and 5.0 ± 0.9 mm, respectively. CTO was present in 49.4% of lesions, with a mean occlusion length of 74.5 ± 100.4 mm. Severe calcification (PACSS grade 4) and severe dissection after pre balloon angioplasty (NHLBI grade D–F) were observed in approximately 26.3% and 14.6% of cases, respectively.

The Combination group had a higher proportion of popliteal artery involvement, a smaller distal reference vessel diameter, and a longer lesion length than the other two groups. In more than 95.0% of procedures, intravascular ultrasound (IVUS) was used (Table 3). Pre-dilation with a non-compliant balloon was performed in 52.7% of cases, and scoring or cutting balloons were used in 28.9%, most frequently in the DCB group. Overall, 89.0% of lesions were treated with DCB angioplasty. Ranger™ was the commonly used DCB, whereas Lutonix™ was used least frequently. DES implantation was performed in 21.6% of cases, and less than half of these received adjunctive DCB therapy. MLA assessed by IVUS differed significantly among the three groups, and was larger in the DES group than in the other groups.

**Table 3:** The detail of revascularization procedure and devices

Variables	Overall (*n* = 273)	DCB group (*n* = 214)	DES group (*n* = 30)	Combination group (*n* = 29)	*P* value
Type of pre-balloon, %					0.02
Semi-compliant balloon	47 (17.2)	30 (14.0)	11 (36.6)	6 (20.6)	
Non-compliant balloon	144 (52.7)	98 (45.7)	13 (43.3)	18 (62.0)	
Chocolate balloon	18 (6.5)	17 (7.9)	1 (3.3)	0 (0)	
Scoring balloon	79 (28.9)	69 (32.1)	5 (16.6)	5 (17.1)	
Diameter of the pre-balloon, mm	5.4 ± 0.7	5.4 ± 0.7	5.3 ± 0.9	5.5 ± 0.7	0.79
Severity of dissection by NHLBI classification, %					0.0007
None	75 (28.1)	61 (28.6)	8 (33.3)	6 (20.6)	
A, B, C	152 (56.9)	128 (60.0)	6 (25.0)	18 (62.0)	
D, E	39 (14.6)	24 (11.2)	10 (41.6)	5 (17.2)	
F	0 (0)	-	-	-	
Residual stenosis ≥ 50% on final angiography, %	19 (6.9)	18 (8.4)	0 (0)	1 (3.4)	0.17
Final device, %					
DCB	243 (89.0)	214 (100)	-	29 (100)	
IN.PACT. Admiral™	90 (32.9)	79 (36.9)	-	12 (41.3)	
Ranger™	146 (53.4)	129 (60.2)	-	16 (55.1)	
Lutonix™	7 (2.5)	6 (2.8)	-	1 (3.4)	
DES	59 (21.6)	-	30 (100)	29 (100)	
Eluvia™	39 (14.2)	-	17 (56.6)	22 (75.8)	
Zilver PTX™	20 (7.3)	-	13 (43.3)	7 (24.1)	
DCB size, mm	5.6 ± 0.6	5.6 ± 0.7	-	5.6 ± 0.4	0.97
DCB length, mm	225 ± 121	230 ± 123	-	188 ± 93	0.13
Stent length, mm	182 ± 93	-	220 ± 101	142 ± 64	0.002
IVUS use, %	260 (95.2)	203 (94.8)	29 (96.6)	28 (96.5)	0.85
MLA assessed by IVUS, mm^2^	14.8 ± 5.3	13.9 ± 4.9	20.4 ± 5.3	15.3 ± 4.6	< 0.0001
Perioperative complication, %	2 (0.7)	1 (0.4)	1 (3.3)	0 (0)	0.20
Contrast agent, mL	93 ± 61	93 ± 63	85 ± 44	101 ± 56	0.63
Procedure time, min	124 ± 83	114 ± 73	168 ± 114	154 ± 95	0.004

Abbreviations: *DCB* drug-coated balloon, *DES* drug-eluting stent, *NHLBI* The National Heart, Lung and Blood Institute, *IVUS* intravascular ultrasound, *MLA* minimum lumen area

**Fig. 2:**
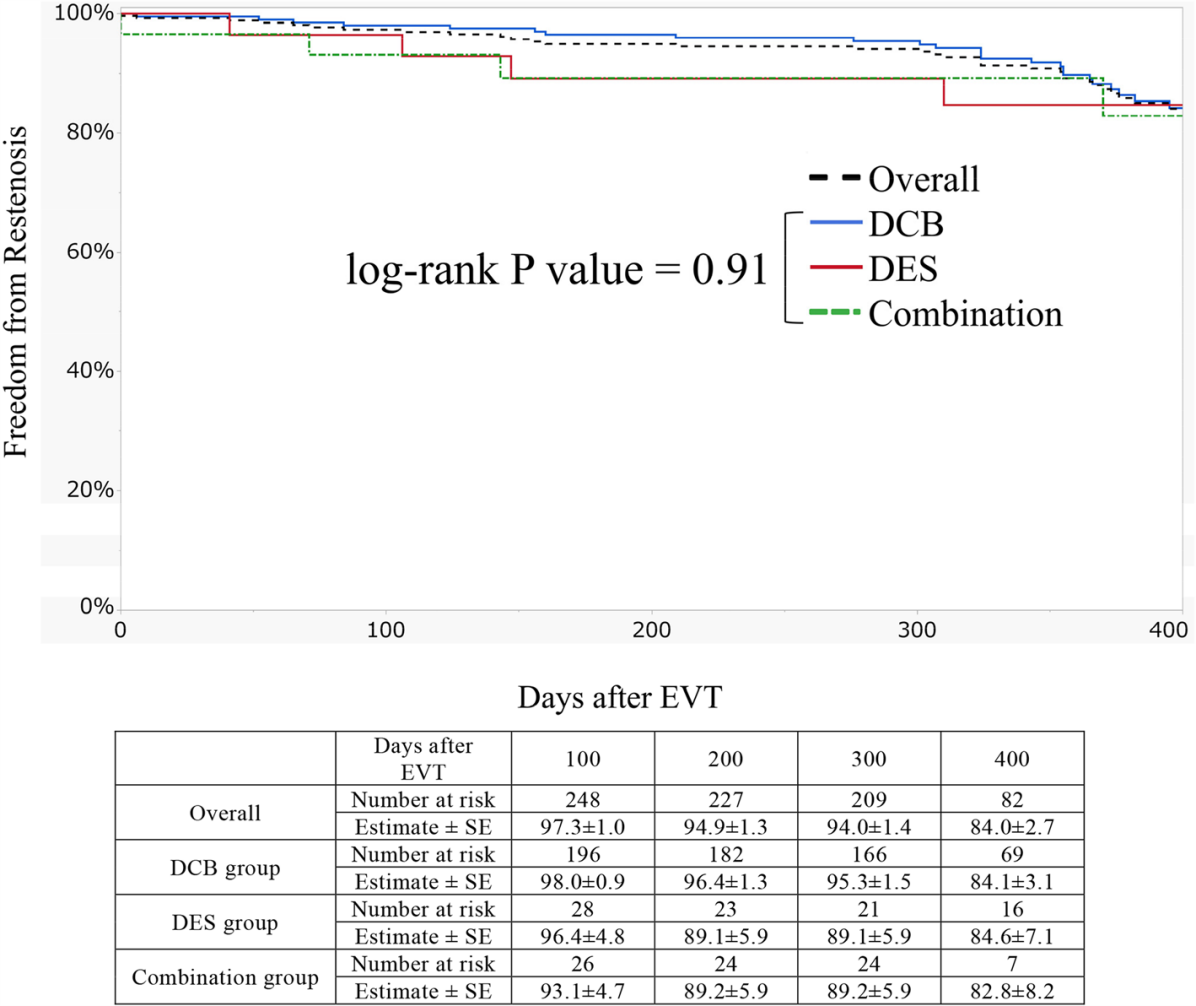
Freedom from restenosis of target lesion by Kaplan–Meier method. Abbreviations: EVT, endovascular therapy; DCB, drug-coated balloon; DES, drug-eluting stent; SE, standard error

**Fig. 3:**
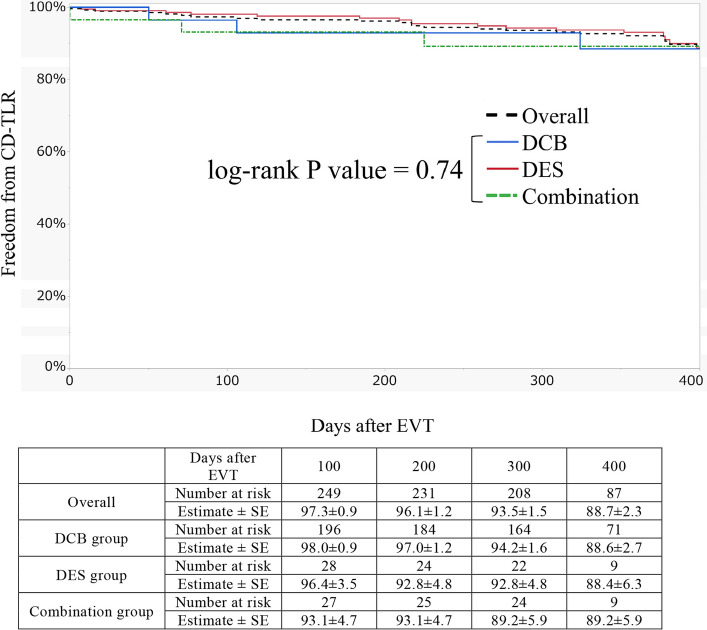
Freedom from clinical-driven target lesion revascularization by Kaplan–Meier method. Abbreviations: CD-TLR, clinical-driven target lesion revascularization; EVT, endovascular therapy; DCB, drug-coated balloon; DES, drug-eluting stent; SE, standard error

**Fig. 4:**
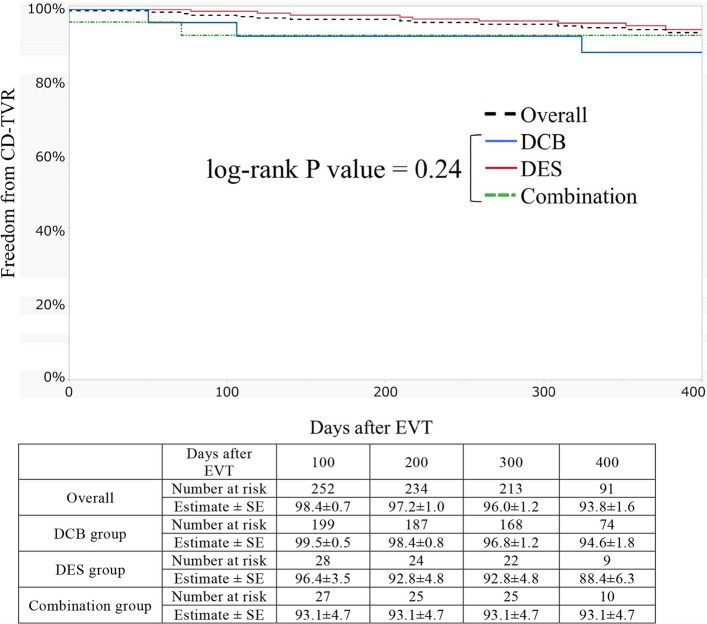
Freedom from clinical-driven target vessel revascularization by Kaplan–Meier method. Abbreviations: CD-TVR, clinical-driven target vessel revascularization; EVT, endovascular therapy; DCB, drug-coated balloon; DES, drug-eluting stent; SE, standard error

Procedural complications occurred in 0.7% of cases, and ≥ 50% residual stenosis on final angiography was observed in 6.9% of cases. Kaplan–Meier estimates demonstrated 1-year freedom from restenosis, CD-TLR, and CD-TVR of 84.0 ± 2.7%, 88.7 ± 2.3%, and 93.8 ± 1.6%, respectively (Figs. 2, 3, and 4). Primary patency did not differ significantly among groups (DCB: 84.1 ± 3.1%, DES: 84.6 ± 7.1%, Combination: 82.8 ± 8.2%; *p* value = 0.91). Similarly, freedom from CD-TLR (88.6 ± 2.7%, 88.4 ± 6.3%, and 89.2 ± 5.9%; *p* value = 0.74), and CD-TVR (94.6 ± 1.8%, 88.4 ± 6.3%, and 93.1 ± 4.7%; *p* value = 0.24) were comparable across treatment strategies.

Univariate Cox regression analysis identified female sex (HR 2.51, 95% CI 1.34–4.66, *p* value = 0.004), CKD on hemodialysis (HR 2.40, 95% CI 1.27–4.55, *p* value = 0.007), CLTI (HR 2.79, 95% CI 1.50–5.18, *p* value = 0.001), history of revascularization (HR 2.64, 95% CI 1.43–4.89, *p* value = 0.002) as factors associated with loss of primary patency (Table 4). In contrast, a larger MLA was associated with patency (HR 0.92, 95% CI 0.86–0.99, *p* = 0.02). In multivariable analysis, history of revascularization (HR 2.88, 95% CI 1.48–5.61, *p* = 0.001) and CLTI (HR 2.09, 95% CI 1.03–4.25, *p* value = 0.04) remained independently associated with loss of primary patency, whereas use of a scoring balloon, severe calcification, a larger MLA, and popliteal artery involvement did not retain statistical significance.

**Table 4:** Association between baseline characteristics and loss of primary patency

Variables	Unadjusted HRs (95%CI), *P* value	Adjusted HRs (95%CI), *P* value
Sex, female	2.51 (1.34–4.66), 0.004	NI
Age, per 10 years	0.72 (0.52–1.02), 0.16	NI
BMI ≦ 18.5 kg/m^2^	1.31 (0.65–2.61), 0.45	NI
Non–ambulatory	1.60 (0.70–3.61), 0.28	NI
Current smoker	0.61 (0.28–1.34), 0.20	NI
CKD on HD	2.40 (1.27–4.55), 0.007	NI
Diabetes mellitus	0.72 (0.37–1.39), 0.34	NI
Heart failure	1.13 (0.56–2.27), 0.71	NI
CLTI	2.79 (1.50–5.18), 0.001	2.09 (1.03–4.25), 0.04
History of revascularization	2.64 (1.43–4.89), 0.002	2.88 (1.48–5.61), 0.001
P2Y12-inhibitor use	0.64 (0.28–1.46), 0.31	NI
Cilostazol use	1.39 (0.66–2.92), 0.39	NI
OAC use	1.89 (0.93–3.87), 0.09	NI
Scoring balloon use	0.70 (0.32–1.53), 0.36	0.61 (0.28–1.33), 0.22
Reference vessel diameter, per − 1.0 mm	1.20 (0.90–1.59), 0.20	NI
Lesion length, per 10 cm	1.01 (0.99–1.04), 0.15	NI
IVUS use	0.99 (0.24–4.15), 0.99	NI
MLA assessed by IVUS, per 1.0 mm^2^	0.92 (0.86–0.99), 0.02	0.93, (0.85–1.00), 0.07
BK poor run off	1.68 (0.91–3.11), 0.09	NI
CTO	1.02 (0.55–1.90), 0.93	NI
PACSS grade 4 vs grade 0–3	2.42 (1.28–4.56), 0.006	1.70, (0.84–3.43), 0.22
POP.A involvement	1.79 (0.95–3.38), 0.06	1.17, (0.59–2.32), 0.65
NHLBI classification D, E, F	1.01 (0.39–2.61), 0.97	NI
Residual stenosis, > 50%	1.86 (0.65––5.27), 0.24	NI
Combination therapy	1.27 (0.49–3.24), 0.62	NI

Abbreviations: *HRs* hazard ratios, *CI* confidence interval, *BMI* body mass index, *CKD* chronic kidney disease, *HD* hemodialysis, *CLTI* chronic limb-threatening ischemia, *OAC* oral anticoagulant, *IVUS* intravascular ultrasound, *MLA* minimum lumen area, *BK* below the knee, *CTO* chronic total occlusion, *PACSS* Peripheral Artery Calcium Scoring System, *POP.A* popliteal artery, *NHLBI* The National Heart, Lung and Blood Institute, *NI* not included

Supplemental Table 1 demonstrates the baseline characteristics of the DCB group and the Combination group after propensity score matching. A total of 26 cases were selected for each group. Matching was performed to balance clinically relevant variables, including lesion length, history of revascularization, reference vessel diameter, and the prevalence of CLTI. However, several variables did not achieve adequate balance, with SMDs remaining above 0.1. Freedom from restenosis (DCB: 71.9 ± 13.0%, Combination: 79.9 ± 9.6%; *p* value = 0.91), CD-TLR (DCB: 85.9 ± 9.9%, Combination: 87.9 ± 6.5%; *p* value = 0.99), and CD-TVR (DCB: 95.4 ± 4.4%, Combination: 92.3 ± 5.2%; *p* value = 0.67) were comparable between two groups after propensity score matching (Supplemental Figs. 2–4).

## Discussion

The present multicenter registry reflects contemporary real-world endovascular practice for FPA lesions and demonstrated favorable 1-year outcomes, with an overall primary patency rate of 84.0% and high freedom from CD-TLR and CD-TVR. These results are consistent with previously reported clinical trials and real-world studies evaluating contemporary DCB and DES technologies, supporting the durability of current endovascular strategies in routine clinical practice [6, 10, 19, 20].

In this cohort, DCB angioplasty was performed in 89.0% of lesions, indicating that a DCB-based approach has become the predominant treatment paradigm in daily clinical practice. Prior studies have identified several predictors of restenosis following DCB treatment, including CLTI, CTO, severe calcification, small vessel diameter, residual stenosis, and popliteal artery involvement [10]. In univariate analysis, several factors, including female sex, CLTI, CKD on hemodialysis, and history of revascularization, were associated with loss of primary patency, reinforcing that clinical severity and lesion complexity remain important determinants of outcome despite advances in paclitaxel-based technology. The limited use of first-generation low-dose DCB devices in this registry may also have contributed to the favorable overall patency rates observed. Moreover, a previous study demonstrated that a larger MLA was associated with improved patency after DCB treatment [21]. In our cohort, a larger MLA was associated with preserved patency in the univariate analysis, although this association did not remain significant after multivariable adjustment.

The Eluvia™ stent was the most frequently used DES in this cohort. Previous reports have demonstrated acceptable 1-year outcomes with Eluvia™, with predictors of restenosis including dialysis, CLTI, history of revascularization, small vessel diameter, CTO, and spot stenting [22]. Notably, lesion length was not significantly associated with patency loss in our study, consistent with earlier findings. It is conceivable that adequate acute luminal gain, particularly in long or complex lesions treated with scaffolding devices, may attenuate the adverse impact of lesion length by minimizing recoil and optimizing drug delivery conditions.

Importantly, the Combination group included more anatomically complex lesions—longer lesion length, a numerically higher prevalence of CTO, smaller reference vessel diameter, and more frequent popliteal artery involvement—yet achieved primary patency comparable to that of DCB and DES monotherapy.

In this registry, all procedures were initiated with a DCB-based strategy, and stenting was added only when pre-dilation resulted in severe dissection or elastic recoil that precluded an acceptable acute result, reflecting routine decision-making in contemporary endovascular practice [23, 24]. Thus, the comparable patency in the Combination group was achieved despite this greater anatomical complexity. Although statistical equivalence cannot be formally established and residual confounding cannot be excluded in this non-randomized registry, no signal toward inferior patency was observed. To further address the baseline imbalance, we performed supplemental propensity score matching between the DCB and Combination groups, and the outcomes remained comparable. Our findings differ from those of Kum et al., [16] who reported inferior patency with stenting; however, that study evaluated the Eluvia stent as a standalone treatment in a single-center retrospective cohort, whereas ours investigated a DCB-based strategy with selective stenting in a multicenter prospective cohort. These differences in study design and treatment strategy may account for the divergent findings.

In multivariable analysis, CLTI and history of revascularization remained independently associated with loss of primary patency, indicating that advanced clinical disease burden remains a key determinant of outcomes despite improvements in contemporary endovascular technologies. These factors likely reflect more aggressive atherosclerotic disease, impaired distal perfusion, and increased lesion complexity, which may limit the durability of endovascular treatment even when contemporary devices and meticulous lesion preparation are applied. Female sex was associated with patency loss in univariate analysis, consistent with previous reports of sex-related differences in disease presentation and outcomes, although the underlying mechanisms warrant further investigation [25, 26].

Taken together, this study provides real-world evidence regarding contemporary device utilization and clinical outcomes in EVT for FPA lesions. These findings suggest that a DCB-based strategy, complemented by selective stent implantation when necessary and supported by meticulous lesion preparation, may represent a practical treatment approach capable of achieving acceptable mid-term outcomes across a wide range of lesion complexity.

## Limitations

This study has several limitations. First, although this was a prospective registry, it was non-randomized and observational, and the treatment strategy was determined at the operator’s discretion. Therefore, residual confounding and selection bias cannot be excluded, particularly in comparisons among the DCB, DES, and Combination groups. Unmeasured confounders may have influenced the observed associations. Moreover, as a supplemental analysis, propensity score matching between the DCB and Combination groups was performed using clinically relevant baseline variables. However, because of the relatively small sample size in the Combination group, several variables did not achieve adequate balance after matching (SMD < 0.1). Therefore, these analyses should be interpreted as exploratory and supplemental.

Second, a relatively high proportion of patients were ambulatory and presented with intermittent claudication. Patients with CLTI often have poor prognosis and physical or social frailty, which may limit regular outpatient follow-up [27–29]. As a result, selection bias may have occurred due to the underrepresentation of more severely frail or non-ambulatory patients.

Third, although predictors of primary patency loss were evaluated using Cox regression analysis, competing risks such as death were not formally accounted for. In addition, angiographic and IVUS findings were assessed at each participating institution without centralized core laboratory adjudication, which may have introduced measurement variability.

Finally, follow-up was limited to 1 year, and longer-term data are necessary to evaluate the durability and safety of each treatment strategy fully.

## Conclusion

In this prospective multicenter registry, a DCB-based strategy was widely adopted for FPA lesions, achieving favorable 1-year outcomes in routine clinical practice. Primary patency was comparable among DCB monotherapy, DES implantation, and combination therapy, despite the latter being applied to more anatomically complex lesions. CLTI and history of revascularization remained independently associated with patency loss, highlighting persistent residual risk in advanced disease. These findings suggest that selective scaffolding within a DCB-based strategy may achieve acceptable patency outcomes even in the presence of more complex baseline lesion characteristics.

## Supplementary Information

Supplementary Material 1: Figs. S1–S3.

Supplementary Material 2: Table S1. 

## Data Availability

The data presented in this article will be shared upon reasonable request to the corresponding author.
