# Supplementary material for: Contemporary outcomes of a DCB-based strategy with selective stent implantation for femoropopliteal artery lesions: results from the REAL-LEAD registry
Source: CVIR Endovasc. 2026 Jul 30;9:93. doi: 10.1186/s42155-026-00744-1 (PMC13424274; doi:10.1186/s42155-026-00744-1)
Supplement: Supplementary file 1 — Supplementary Material 1: Figs. S1–S3. [file 42155_2026_744_MOESM1_ESM.zip › Supplemental Figure1R2.docx]

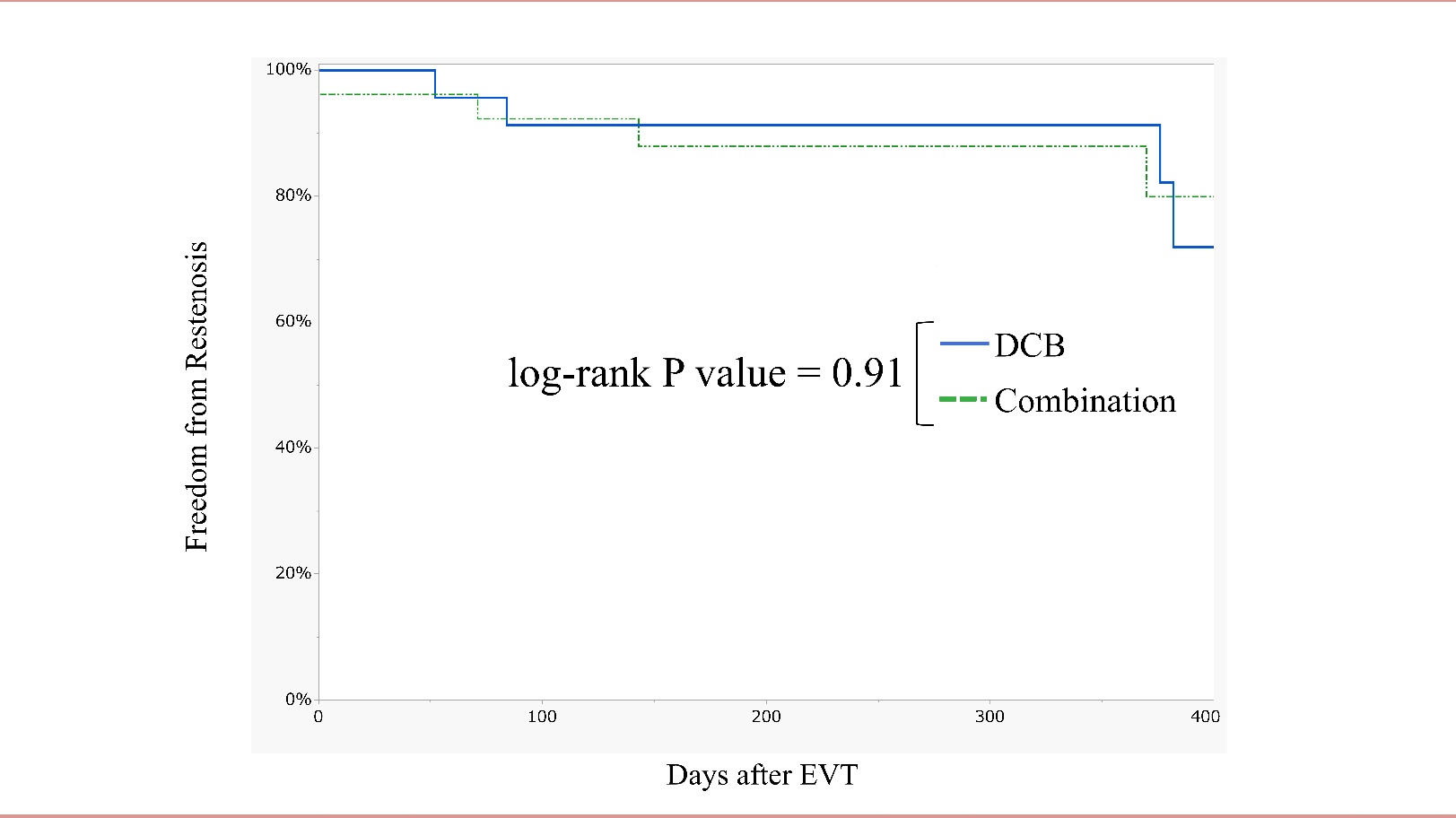


|  | Days after EVT | 100 | 200 | 300 | 400 |
| --- | --- | --- | --- | --- | --- |
| DCB group | Number at risk | 22 | 18 | 16 | 6 |
|  | Estimate ± SE | 91.3±5.8 | 91.3±5.8 | 91.3±5.8 | 71.9±13.0 |
| Combination group | Number at risk | 23 | 21 | 21 | 6 |
|  | Estimate ± SE | 92.3±5.2 | 87.9±6.5 | 87.9±6.5 | 79.9±9.6 |

Supplemental Figure1
