# Supplementary material for: Contemporary outcomes of a DCB-based strategy with selective stent implantation for femoropopliteal artery lesions: results from the REAL-LEAD registry
Source: CVIR Endovasc. 2026 Jul 30;9:93. doi: 10.1186/s42155-026-00744-1 (PMC13424274; doi:10.1186/s42155-026-00744-1)
Supplement: Supplementary file 2 — Supplementary Material 2: Table S1. [file 42155_2026_744_MOESM2_ESM.docx]

Supplemental Table 1. The Baseline of Patient and Lesion Characteristics after Propensity Score Matching.

| variables | DCB group | Combination group | SMD | P value |
| --- | --- | --- | --- | --- |
| Matched patient | 26 | 26 | - | - |
| Age (years) | 72.0±8.4 | 74.0±12.4 | 0.19 | 0.48 |
| Sex (female) | 7 (26.9) | 9 (34.6) | 0.16 | 0.76 |
| BMI (kg/m^2^) | 23.1±4.8 | 21.8±3.3 | 0.31 | 0.26 |
| Current smoker | 8 (30.7) | 4 (15.3) | 0.37 | 0.32 |
| Fully ambulant | 20 (76.95) | 18 (69.2) | 0.17 | 0.75 |
| Hypertension | 25 (96.1) | 21 (80.7) | 0.49 | 0.19 |
| Dyslipidemia | 21 (80.7) | 17 (65.3) | 0.35 | 0.34 |
| Diabetes mellitus | 13 (50.0) | 18 (69.2) | 0.39 | 0.25 |
| Coronary artery disease | 12 (46.1) | 14 (53.8) | 0.15 | 0.78 |
| CKD on hemodialysis | 10 (38.4) | 8 (30.7) | 0.16 | 0.77 |
| Heart failure | 7 (26.9) | 11 (42.3) | 0.32 | 0.38 |
| CLTI | 18 (69.2) | 16 (61.5) | 0.16 | 0.77 |
| Creatinine(mg/dL) | 0.9±0.4 | 0.9±0.3 | 0.09 | 0.78 |
| estimated GFR  (ml/min/1.73m^2^) | 63.4±21.5 | 63.7±25.2 | 0.01 | 0.97 |
| LDL cholesterol (mg/dL) | 75.9±24.4 | 94.7±24.6 | 0.79 | 0.009 |
| HbA1c (%) | 6.2±1.1 | 6.3±1.0 | 0.16 | 0.56 |
| Aspirin | 22 (84.6) | 15 (57.6) | 0.62 | 0.06 |
| P2Y12 inhibitor | 23 (88.4) | 21 (80.7) | 0.21 | 0.70 |
| Cilostazol | 2 (7.6) | 3 (11.5) | 0.13 | 1.00 |
| Oral anticoagulant | 2 (7.6) | 8 (30.7) | 0.61 | 0.07 |
| Statin | 18 (69.2) | 20 (76.9) | 0.17 | 0.75 |
| de novo lesion, % | 22 (84.6) | 18 (69.2) | 0.37 | 0.32 |
| history of EVT, % | 5 (19.2) | 7 (26.9) | 0.18 | 0.74 |
| POP.A involvement, % | 18 (69.2) | 15 (57.6) | 0.32 | 0.56 |
| lesion length, mm | 248±116 | 274±89 | 0.25 | 0.37 |
| distal reference diameter, mm | 4.8±1.0 | 4.8±1.0 | 0.07 | 0.79 |
| BK poor run off  (0 or 1), % | 8 (30.7) | 11 (42.3) | 0.24 | 0.56 |
| CTO, % | 13 (50.0) | 15 (57.6) | 0.15 | 0.57 |
| CTO length, mm | 80±100 | 74±92 | 0.06 | 0.82 |
| severe calcification  (PACSS grade 4), % | 12 (46.1) | 10 (38.4) | 0.15 | 0.77 |
| TASC Ⅱ classification  (C or D), % | 15 (57.6) | 17 (65.3) | 0.15 | 0.77 |

The items of “age”, “BMI”, “lesion length”, and “CTO length” were summarized as mean values with standard deviations. The laboratory data were summarized as mean values with standard deviations and counts. Other data were summarized as counts and percentages. Abbreviations; DCB, drug-coated balloon; SMD, standardized mean difference; BMI, body mass index; CKD, chronic kidney disease; CLTI, chronic limb-threatening ischemia; estimated GFR, estimated glomerular filtration rate is calculated using the Cockcroft-Gault formula; LDL, low-density lipoprotein; HbA1c, hemoglobinA1c; EVT, endovascular therapy; POP.A, popliteal artery; BK, below the knee; CTO, chronic total occlusion; PACSS, Peripheral Artery Calcification Scoring System; TASC, Trans-Atlantic Inter-Society Consensus.
